# Fractional-Order Approximation of PID Controller for Buck–Boost Converters

**DOI:** 10.3390/mi12060591

**Published:** 2021-05-21

**Authors:** Allan G. S. Sánchez, Josué Soto-Vega, Esteban Tlelo-Cuautle, Martín Antonio Rodríguez-Licea

**Affiliations:** 1 CONACYT—Instituto Tecnológico de Celaya, Guanajuato 38010, Mexico; martin.rodriguez@itcelaya.edu.mx; 2 Instituto Tecnológico de Celaya, Guanajuato 38010, Mexico; m2003021@itcelaya.edu.mx; 3 Department of Electronics, INAOE, Puebla 72840, Mexico; etlelo@inaoep.mx

**Keywords:** fractional-order PID controller, DC–DC converters, Non-minimum phase systems, experimental validation

## Abstract

Viability of a fractional-order proportional–integral–derivative (PID) approximation to regulate voltage in buck–boost converters is investigated. The converter applications range not only to high-power ones but also in micro/nano-scale systems from biomedicine for energy management/harvesting. Using a classic closed-loop control diagram the controller effectiveness is determined. Fractional calculus is considered due to its ability at modeling different types of systems accurately. The non-integer approach is integrated into the control strategy through a Laplacian operator biquadratic approximation to generate a flat phase curve in the system closed-loop frequency response. The controller synthesis considers both robustness and closed-loop performance to ensure a fast and stable regulation characteristic. A simple tuning method provides the appropriate gains to meet design requirements. The superiority of proposed approach, determined by comparing the obtained time constants with those from typical PID controllers, confirms it as alternative to controller non-minimum phases systems. Experimental realization of the resulting controller, implemented through resistor–capacitor (RC) circuits and operational amplifiers (OPAMPs) in adder configuration, confirms its effectiveness and viability.

## 1. Introduction

DC–DC (direct current) conversion is one of the most studied and applied functionalities from power electronics. DC–DC elementary conversion modes step down and up the converter input voltage using power semiconductor devices, operated with high-speed control, to produce the well-known buck and boost topologies, respectively. By cascading both elementary conversion modes the buck–boost topology is obtained. With the same quantity of elements, the resulting converter produces a smaller or greater output voltage than its input power source, with inverse polarity.

The versatility of buck–boost converter to transform its supply voltage into a higher/lower output makes it an alternative for applications requiring DC voltage regulation, ranging from light-emitting diode (LED) lighting [1] to renewable energy sources [2,3], microgrids [4], and battery charging [5,6], to mention the most relevant. Biomedicine applications [7,8,9] take special relevance due to their impact in human’s health, since the need for appropriate energy management/harvesting/storage strategies to be applied in micro/nano-scale is one of the main drawbacks of emerging cardiac technologies, for instance. Some of the most recent and significant results on strategies to regulate voltage in a buck–boost converter are the following: in [10], a deep learning-based approach was used to stabilize voltage in the converter. A sliding mode-based observer combined with an optimization algorithm, a deep reinforcement learning technique, and a neural network were suggested to estimate converter unknown dynamics, while controller gains were adjusted online. Good transient behavior and output-robust stabilization against reference changes were the major improvements. In [11], an intelligent control with metaheuristic optimization was proposed. A fish-swarm algorithm is used to optimally tune a PI controller to regulate voltage in the converter. The simplicity of the approach is the strongest advantage of this contribution. The effectiveness of the controller was determined experimentally, where a fast-tracking characteristic was the main improvement. A fuzzy logic controller to stabilize voltage through a buck–boost converter in a turbine generation unit was proposed in [12]. Takagi–Sugeno-type rules were employed due to their wider range for gain variations and versatility. Even though authors compare open-loop vs. closed-loop performance to determine effectiveness, the latter exhibited acceptable time constants, and good tracking performance.

A passivity-based control was suggested in [13] to stabilize voltage fed to a microgrid. The proposed strategy achieved Lyapunov asymptotic stability through a state-feedback control law, which required an online invariance and immersion power estimator. In spite of authors compared their results with a pure PI to determine controller effectiveness, acceptable tracking characteristic, good transient performance and negligible steady-state error for both conversion modes were the improvements. With a similar approach but targeting a different application, a passivity-based controller with active disturbance rejection was proposed in [14]. A generalized proportional integral observer was used to provide accurate estimations to the controller. Good tracking characteristic and disturbances rejected effectively are the main contribution.

In [15], a modified sliding mode controller was proposed. The control strategy was divided into two portions, a linear approach based on a PI controller for voltage control loop and a nonlinear one for current loop based on hysteresis. The resulting regulated output voltage described a smooth response with overshoot absence, acceptable robustness against load variations, and good tracking performance. In [16], a predictive control approach was proposed. The authors combined a quadratic programming optimization algorithm, to predict the control signal at every sampling time, with a predictive controller, to consider load variations in the model, thus ensuring robustness and stability. The appropriate control law was generated by predicting the future behavior of the plant, resulting in a fast response, minimum overshoot and good tracking characteristic. On the other hand, due to the inherent closed-loop instability of non-minimum phase systems, PI/PID controllers are used in combination with some of the above-described techniques or some optimization algorithms. The above derived in control strategies, although efficient, with high computational/implementation complexity [17,18,19].

In this paper, a fractional-order PID controller approximation to regulate voltage in a buck–boost converter is proposed. In addition to the accuracy modeling real systems, robustness against parameter variations, and noise-level reduction through lower-order derivatives from fractional calculus, exploring its effectiveness controlling non-minimum phases systems is the main reason to consider a non-integer approach in the control strategy. The controller synthesis is achieved through a biquadratic module that exhibits a flat phase response. Its design considers both robustness and closed-loop performance, while a simple tuning method allows us to determine appropriate gains to achieve design requirements. The controller structure to operate in both conversion modes is generalized to generate its electrical representation directly. Numerical and experimental results are provided to corroborate effectiveness of the proposed approach.

The paper is organized as follows: in Section 2 preliminaries on buck–boost converter and the methodology to approximate fractional-order differentiator are provided. In Section 3 the algorithm to synthesize the controller is described step by step. Numerical simulations, including a comparison with a typical PID controller, a generalization of the synthesized controller, and experimental results of the obtained electrical arrangement are presented in this section as well. A discussion on the most significant results, including future work and some conclusions/remarks, are given in Section 4 and Section 5, respectively.

## 2. Materials and Methods

In this section, the necessary preliminaries on buck–boost converter, the small-signal linearization of its model, and fractional-order approximation of Laplacian operator are briefly described.

### 2.1. Buck–Boost Converter Model

The DC–DC buck–boost converter was derived from the combination of elementary converters buck and boost. The resulting configuration can provide an output voltage of inverse polarity, either greater or smaller than the input voltage, with the same amount of elements.

**Figure 1:**
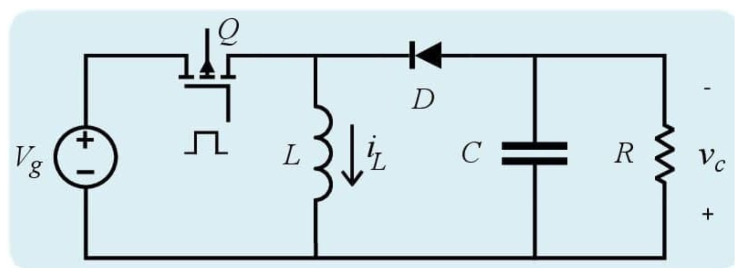
Electrical diagram of the DC–DC (direct current) buck–boost converter.

Figure 1 shows the electrical diagram of the buck–boost converter. Assuming ideal components and continuous conduction mode (CCM), the averaged model of the buck–boost converter is obtained as follows [20],

(1) $$\begin{array}{ccc} L\frac{di_{L}}{\mathrm{dt}} & = & V_{g}\bar{D}+\left(1-\bar{D}\right)v_{C},\\ C\frac{dv_{C}}{\mathrm{dt}} & = & -\left(1-\bar{D}\right)i_{L}-\frac{v_{C}}{R}, \end{array}$$

where $\bar{D}\in \left[0,1\right]$, $V_{g}$ is the DC power supply, *L*, *C* and *R* are the inductance, capacitance and the resistance, respectively.

Linearization of (1) is performed through the small-signal technique, which consists of perturbing the original model signals to generate its DC and alternating current (AC) components [21]. The resulting AC component will be the linearized small-signal average model of the buck–boost converter, whose state space representation $\dot{x}=Ax+Bu$ and y=Cx around the equilibrium point $\left[i_{L},\;v_{C}\right]=\left[V_{g}\bar{D}/\left(R{\left(1-\bar{D}\right)}^{2}\right),\;V_{g}\bar{D}/\left(1-\bar{D}\right)\right]$ is given by [21],

(2) $$\left[\begin{array}{c} {\dot{\hat{i}}}_{L}\\ {\dot{\hat{v}}}_{C} \end{array}\right]=\left[\begin{array}{cc} 0 & \frac{\left(1-\bar{D}\right)}{L}\\ \frac{-(1-\bar{D})}{C} & \frac{-1}{RC} \end{array}\right]\left[\begin{array}{c} {\hat{i}}_{L}\\ {\hat{v}}_{C} \end{array}\right]+\left[\begin{array}{cc} \frac{V_{g}}{L(1-\bar{D})} & \frac{\bar{D}}{L}\\ \frac{V_{g}\bar{D}}{RC{\left(1-\bar{D}\right)}^{2}} & 0 \end{array}\right]\left[\begin{array}{c} \hat{\bar{d}}\\ {\hat{v}}_{g} \end{array}\right],$$

and

(3) $$y=\left[\begin{array}{cc} 0 & 1 \end{array}\right]\left[\begin{array}{c} {\hat{i}}_{L}\\ {\hat{v}}_{C} \end{array}\right],$$

where ${\hat{i}}_{L}$, ${\hat{v}}_{C}$, $\hat{\bar{d}}$ and ${\hat{v}}_{g}$ are the perturbation terms of $i_{L}$, $v_{C}$, $\bar{D}$ and $V_{g}$, respectively.

The transfer function of buck–boost converter $G_{p}\left(s\right)$ will be given by the relation $\hat{\bar{d}}$-to-${\hat{v}}_{c}$ as follows,

(4) $$G_{p}\left(s\right)=C{\left(sI-A\right)}^{-1}B_{1},$$

where $B_{1}={\left[\begin{array}{cc} \frac{V_{g}}{L(1-\bar{D})} & \frac{Vg\bar{D}}{RC{\left(1-\bar{D}\right)}^{2}} \end{array}\right]}^{T}$. Thus, the system transfer function will be given by,

(5) $$G_{p}\left(s\right)=\frac{\left(\frac{V_{g}\bar{D}}{RC{\left(1-\bar{D}\right)}^{2}}\right)s-\left(\frac{V_{g}}{CL}\right)}{s^{2}+\left(\frac{1}{RC}\right)s+\frac{{\left(1-\bar{D}\right)}^{2}}{CL}},$$

which has a right-half plane (RHP) zero, thus it is a non-minimum phase transfer function. The buck–boost converter transfer function (5) can be divided into factors as follows,

(6) $$G_{p}\left(s\right)=G_{pm}\left(s\right)G_{nm}\left(s\right),$$

where

(7) $$G_{pm}\left(s\right)=\frac{\left(\frac{V_{g}\bar{D}}{RC{\left(1-\bar{D}\right)}^{2}}\right)\left(s+\frac{R{\left(1-\bar{D}\right)}^{2}}{L\bar{D}}\right)}{s^{2}+\left(\frac{1}{RC}\right)s+\frac{{\left(1-\bar{D}\right)}^{2}}{CL}},$$

(8) $$G_{nm}\left(s\right)=\frac{s-\frac{R{\left(1-\bar{D}\right)}^{2}}{L\bar{D}}}{s+\frac{R{\left(1-\bar{D}\right)}^{2}}{L\bar{D}}},$$

are the minimum phase and normalized non-minimum phase parts of $G_{p}\left(s\right)$, respectively. Please note that $G_{nm}\left(s\right)$ is an all-pass system, i.e., $|G_{nm}\left(j\omega \right)|=1$, thus, the converter dynamic is given by the minimum phase part $G_{pm}\left(s\right)$, which will be considered to be the uncontrolled plant. The non-minimum phase part commonly introduces a delay, but it is also responsible for the output polarity inversion.

In the following, the methodology to approximate the non-integer PID controller is described.

### 2.2. Fractional-Order Approximation of Laplacian Operator

In this section, the approximation of the fractional-order Laplacian operator is described.

The integro-differential operator $s^{\pm \alpha }$ can be approximated as follows [22,23],

(9) $$s^{\alpha }\approx T\left(\frac{s}{\omega_{c}}\right)=\frac{a_{0}{\left(\frac{s}{\omega_{c}}\right)}^{2}+a_{1}\left(\frac{s}{\omega_{c}}\right)+a_{2}}{a_{2}{\left(\frac{s}{\omega_{c}}\right)}^{2}+a_{1}\left(\frac{s}{\omega_{c}}\right)+a_{0}},\;\;\;\;\;0<\alpha <1,$$

which is a biquadratic module that exhibits a flat phase response, where $\omega_{c}$ stands for the center frequency and $a_{0}$, $a_{1}$, $a_{2}$ are alpha-dependent real constants defined as follows,

(10) $$\begin{array}{ccc} a_{0} & = & \alpha^{\alpha }+3\alpha +2,\\ a_{2} & = & \alpha^{\alpha }-3\alpha +2,\\ a_{1} & = & 6\alpha \;\tan\frac{(2-\alpha )\pi }{4}. \end{array}$$

By assuming $\omega =\omega_{c}$ and considering constants (10), the integro-differential operator (9) will be described as

(11) $$s^{\alpha }\approx T\left(\frac{j\omega_{c}}{\omega_{c}},\alpha \right)=\frac{\left(a_{2}-a_{0}\right)+ja_{1}}{-\left(a_{2}-a_{0}\right)+ja_{1}}=\frac{-6\alpha +j6\alpha \tan\frac{(2-\alpha )\pi }{4}}{6\alpha +j6\alpha \tan\frac{(2-\alpha )\pi }{4}},$$

whose phase contribution will be given by $\arg\left\{T\left(j1,\alpha \right)\right\}=-2\tan^{-1}\left(\tan\frac{(2-\alpha )\pi }{4}\right)$. Thus, the phase contribution of approximation (9) will be given by [22,23],

(12) $$\arg\{T\left(s/\omega_{c}\right){|}_{j\omega_{c}}\}=\pm \alpha \pi /2,$$

which means that depending on the value of α, the biquadratic approximation of $s^{\pm \alpha }$ contributes with $0<\arg\left\{T\left(s/\omega_{c}\right){|}_{j\omega_{c}}\right\}<\pm 90^{\circ }$.

Equation (9) will behave as a fractional-order differentiator around $\omega_{c}$ as long as $a_{0}>a_{2}>0$, i.e., $\arg\{T\left(s/\omega_{c}\right)\}>0$. Conversely, the effect of fractional-order integrator can be produced by ensuring that $0<a_{0}<a_{2}$, which produces $\arg\{T\left(s/\omega_{c}\right){|}_{j\omega_{c}}\}<0$. The latter is consistent with the location of zeros/poles of (9) in the complex plane, where zeros lead poles for $a_{0}>a_{2}$ and poles lead zeros for $a_{0}<a_{2}$, which confirms derivative/integral effects.

**Figure 2:**
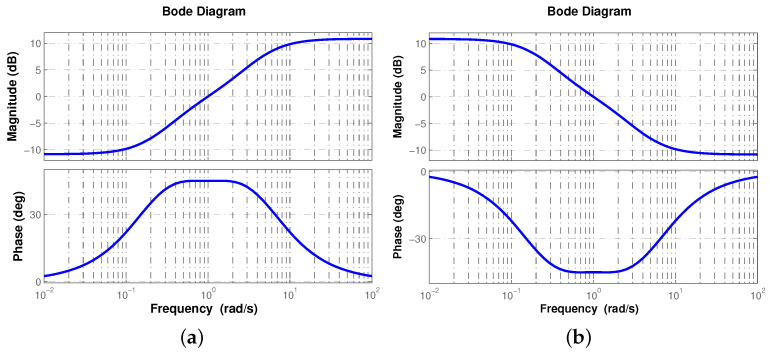
Frequency response of $s^{\pm 0.5}$, where $\arg\left\{T\left(j1\right)\right\}=\pm \alpha \pi /2=\pm 45^{\circ }$ for both (**a**) derivative and (**b**) integral effect of (9).

In Figure 2 the frequency response of $s^{\pm 0.5}$ is shown, where $a_{0}=4.2071$, $a_{1}=7.2427$ and $a_{2}=1.2071$ to ensure derivative (Figure 2a) and integral (Figure 2b) effects.

The synthesis of controller structure, using the fractional-order approximation of Laplacian operator, is described in the following section.

### 2.3. Synthesis of Fractional-Order PID Approximation

The standard representation of a PID controller $G_{c}\left(s\right)=k_{p}\left(1+1/\left(T_{i}s\right)+T_{d}s\right)$ was modified to consider the non-integer approach in its integral/derivative modes. The fractional-order PI${}^{\alpha }$D${}^{\mu }$ structure is a special case of PID controller with additional degrees of freedom which is described as follows [24]

(13) $$G_{c}\left(s\right)=k_{p}\left(1+\frac{1}{T_{i}s^{\alpha }}+T_{d}s^{\mu }\right),$$

where 0<α,μ<1, $k_{p}$ is the proportional gain, $T_{i}$ and $T_{d}$ are the integral and derivative time constants, respectively.

Different optimization strategies have suggested that $T_{i}$ and $T_{d}$ are related through $T_{i}=\eta T_{d}$, where η is a constant. The first suggestion derived in η=4 aiming to achieve a compromise between controller performance and its viability to be implemented [25]. Motivated by the need to ensure unique solutions of (13), some other results showed that smaller values of η produced significant improvements [25,26]. By slightly modifying (13) and by setting α=μ, η=1, (13) can be expressed as follows,

(14) $$G_{c}\left(s\right)=\frac{k_{c}{\left(T_{i}s^{\alpha }+1\right)}^{2}}{s^{\alpha }},$$

which directly simplifies the PID structure through a perfect square trinomial [27], where $k_{c}=k_{p}/T_{i}$.

The phase of the plant to be controlled $\phi_{pm}$, the controller phase $\phi_{c}$ and phase margin $\phi_{m}$ are related through $\phi_{c}+\phi_{pm}=-\pi +\phi_{m}$ at the phase crossover frequency $\omega_{pc}$, which implies that $\phi_{c}=\phi_{m}-\pi -\phi_{pm}$, thus,

(15) $$\alpha =\frac{\left(-\pi -\phi_{pm}+\phi_{m}\right)}{\left(\pi /2\right)}.$$

In the next section, the fractional-order PID controller approximation is validated numerically and experimentally. A generalization of its structure for buck and boost modes of the converter is derived as well.

## 3. Results

In this section, the methodology to synthesize a fractional-order approximation of a PID controller is described step by step. The generalization of its structure for buck and boost conversion modes is derived. Numerical and implementation results are provided to show its effectiveness.

### 3.1. Control Design and Numerical Results

The suggested algorithm to synthesize the approximation of the fractional-order PID (FOPID) controller for the buck–boost converter comprises the following steps:

1. Consider buck–boost converter transfer function divided into minimum and non-minimum phase parts.
2. Think on the minimum phase transfer function $G_{pm}\left(s\right)$ as the plant to be controlled.
3. Determine uncontrolled plant phase $\phi_{pm}$ and the phase margin $\phi_{m}$.
4. Compute the required controller fractional-order α through (15).
5. Compute fractional-order approximation $s^{\alpha }$ through (9).
6. Generate controller structure $G_{c}\left(s\right)$ as a function of integral time constant $T_{i}$ and gain $k_{c}$.
7. Determine $T_{i}$ and $k_{c}$ values that produce the required effect.
8. Determine regulation/tracking performance of the closed-loop response.

Since buck–boost converter operates in both elementary conversion modes and considering that its transfer function is one of varying parameters, controller design is developed under the following conditions: buck–boost converter transfer function (5) and (6), whose parameter values are shown in Table 1, desired stability margins of $g_{m}\geq 10$ dB, $30^{\circ }\leq \phi_{dm}\leq 60^{\circ }$ and equilibrium points generated with the average duty cycle $\bar{D}=0.375$ and $\bar{D}=0.583$ for buck and boost conversion modes to produce an output voltage of $V_{o}=15$ V and $V_{o}=35$ V, respectively.

By considering the described conditions, the minimum phase transfer function $G_{pm}\left(s\right)$ of buck–boost converter is linearized around equilibrium points $\left[i_{L},\;v_{C}\right]=\left[2.4,\;-15\right]$ for buck conversion mode and $\left[i_{L},\;v_{C}\right]=\left[8.4,\;-35\right]$ for boost conversion mode. In Table 2, computation of phase margin $\phi_{m}$, uncontrolled plant phase $\phi_{pm}$, and fractional-order α for buck and boost conversion modes are provided.

**Figure 3:**
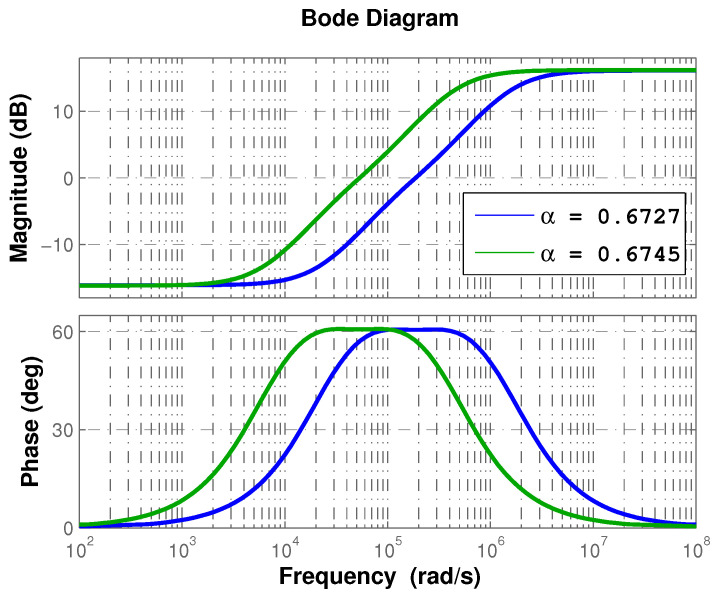
Frequency response of fractional-order approximation for buck/boost conversion modes.

Therefore, the controller phase contributions are $-60.7^{\circ }$ and $-60.5^{\circ }$ for buck and boost conversion modes, respectively. Please note that the algorithm for controller design provides a very similar result for both conversion modes up to this point. However, it should be kept in mind that the transfer function is of varying parameters; thus, its frequency response changes depending on the equilibrium point considered in the linearization. In Figure 3, the fractional-order approximations that are generated with the computed values of α are shown. One can see their similarity in shape and phase contribution, but they differ in their frequency band.

**Figure 4:**
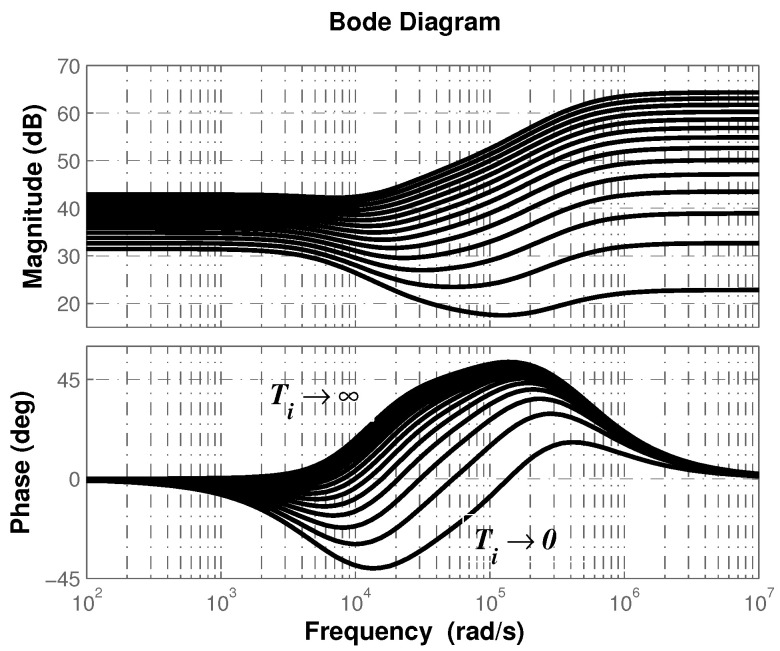
Transition between integral and derivative effect as function of $T_{i}$.

The controller $G_{c}\left(s\right)$ can be obtained by manipulating (9) as $s^{\alpha }\approx T\left(s\right)\equiv N\left(s\right)/D\left(s\right)$ and then substituting in (14), thus the controller structure will be given by,

(16) $$G_{c}\left(s\right)=k_{c}\frac{{\left(T_{i}N\left(s\right)+D\left(s\right)\right)}^{2}}{N(s)D(s)},$$

from which one concludes that controller effect can be varied between integral and derivative depending on the value of $T_{i}$. In Figure 4 the transition between both effects are shown. As can be seen, integral (derivative) effect is achieved as $T_{i}\to 0$ ($T_{i}\to \infty$).

By setting $T_{i}=0.001$ and $k_{c}=3$, the controller $G_{c}\left(s\right)$ produces the necessary effect to fulfill the required stability margins in both conversion modes. The resulting controller structure for converter in Figure 1 is given by,

(17) $$G_{c}\left(s\right)=k\frac{s^{4}+\rho_{1}s^{3}+\rho_{2}s^{2}+\rho_{3}s+\rho_{4}}{s^{4}+\psi_{1}s^{3}+\psi_{2}s^{2}+\psi_{3}s+\psi_{4}},$$

whose parameters are listed in Table 3.

**Figure 5:**
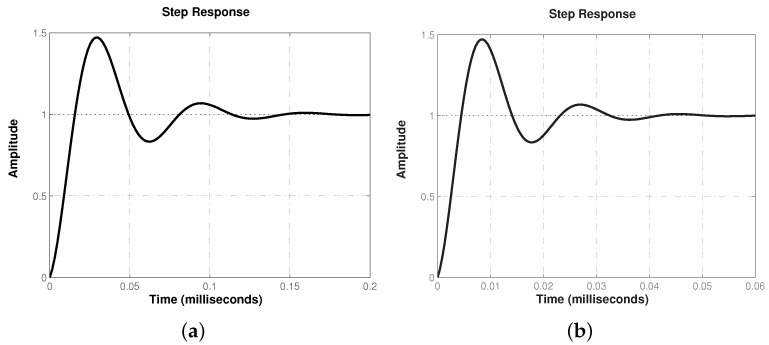
Closed-loop step response for both (**a**) buck and (**b**) boost conversion modes of converter in Figure 1.

The step response of the closed-loop system will allow us to determine the effectiveness of the synthesized controller. In Figure 5, the regulation capacity of the proposed controller can be confirmed. One can see that the response exhibits a fast and stable tracking characteristic for both conversion modes, which can be corroborated quantitatively through performance parameters in Table 4, column 2.

Although regulation velocity of the response is acceptable, the proposed controller performs different depending on the selected conversion mode. These differences are attributed to the operating frequency band of the approximation, being the one for boost conversion mode of a higher frequency range, as shown in Figure 3.

A comparison of the fractional-order PID approximation with a typical PID controller allows us to determine that the former outperforms the latter. By using MatLab algorithm to tune PID controller, targeting 60${}^{\circ }$ phase margin and a compromise between robustness and performance, typical PID controllers were tuned, resulting ${\left[k_{p},\;T_{i},\;T_{d}\right]}_{\mathrm{buck}}=\left[0.068,\;3.85\times 10^{-5},\;9.92\times 10^{-9}\right]$ and ${\left[k_{p},\;T_{i},\;T_{d}\right]}_{\mathrm{boost}}=\left[0.021,\;2.59\times 10^{-5},\;8.04\times 10^{-9}\right]$ for both conversion modes. Performance of inter-order PID controller is quantified in Table 4 column 3. By comparing columns 2 and 3, the proposed approach superiority can be determined through time constants since the ones obtained with the approximation of fractional-order PID controller are much smaller. Although typical PID achieves the output regulation with zero steady-state error, it took longer in both conversion modes to reach the reference value.

To determine if a typical PID structure can equalize performance of the proposed approach and considering that an increase in performance reduces robustness and vice versa, a second option of PID controller was tuned with a less restrictive phase margin aiming to obtain performance improvement, resulting ${\left[k_{p},\;T_{i},\;T_{d}\right]}_{\mathrm{buck}}=\left[0.034,\;1.51\times 10^{-5},\;3.78\times 10^{-6}\right]$ and ${\left[k_{p},\;T_{i},\;T_{d}\right]}_{\mathrm{boost}}=\left[0.123,\;1.37\times 10^{-6},\;3.43\times 10^{-7}\right]$ for both conversion modes. In Table 4, column 4 performance parameters produced by the second PID controller are provided. Please note that despite the increase in performance, the second option of integer-order PID controller produces time constants that are not competitive with those of the proposed approach.

**Figure 6:**
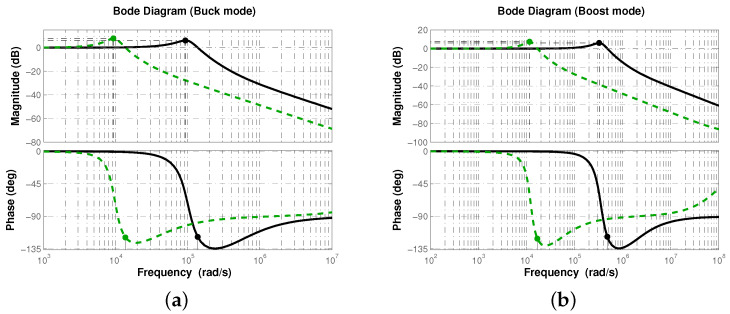
Frequency response of closed-loop system for both (**a**) buck and (**b**) boost conversion modes.

The comparison is moved to the frequency domain to corroborate the stability margins and determine the effect of controller on the magnitude/phase curves of the closed-loop response. In Figure 6, frequency response of closed-loop system with fractional-order PID and typical PID controllers, operating in buck and boost conversion modes, are shown. One can see that both controllers were able to achieve the desired phase margin. Note the shape similarity of the magnitude curves. They both have their peaks around the same value, which corroborates that overshoot is similar for both controllers. The operating frequency band is wider for the system controlled with the fractional-order PID approximation. The latter is consistent with the response velocity measured for system controlled with the proposed controller, since the wider the bandwidth the shorter the rising time, due to the higher-frequency signals pass through the system more easily.

**Figure 7:**
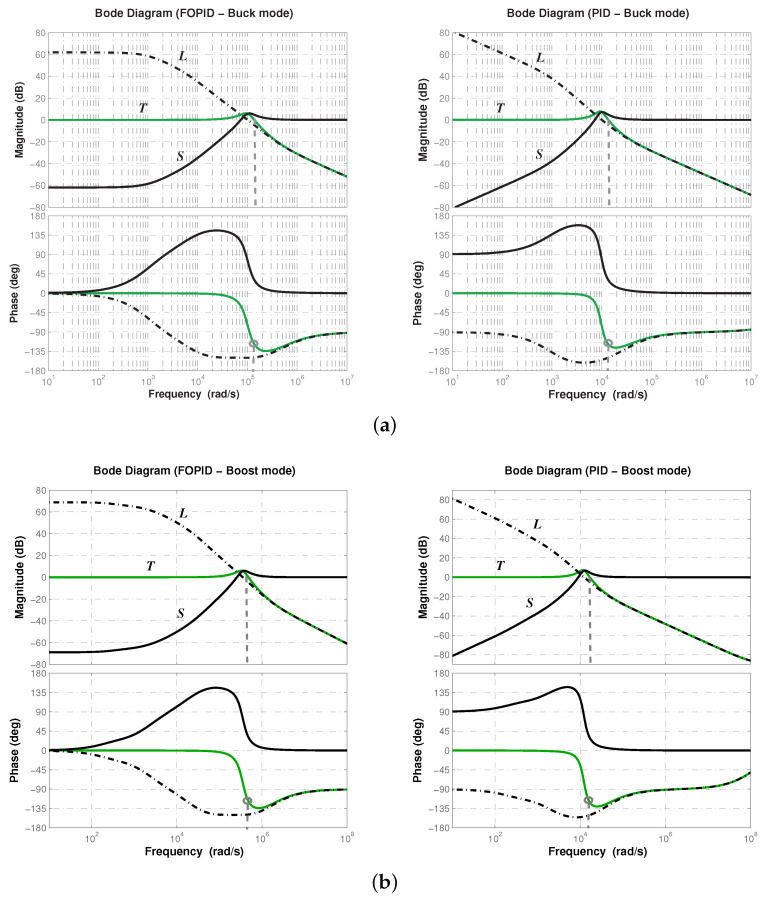
Frequency response of sensitivity *S*, complementary sensitivity *T* functions and loop gain *L* for both (**a**) buck and (**b**) boost conversion modes.

Lastly, frequency response of the sensitivity and complementary sensitivity functions allows us to determine the robustness of the controller through its disturbance and noise rejection characteristics. Recalling that sensitivity function $S=1/(1+G_{p}G_{c})$, and complementary sensitivity function $T=G_{p}G_{c}/\left(1+G_{p}G_{c}\right)$ determine how disturbances/perturbations and noise affect respectively the output, it is expected the controller $G_{c}$ to produce a curve with attenuation in low frequencies for *S* and a curve with attenuation in high ones for *T* ([28], Chap. 4). In Figure 7, frequency response of sensitivity *S* and complementary sensitivity *T* functions is shown for both conversion modes, where $L=G_{p}G_{c}$ is the loop gain, when using fractional-order PID and typical PID controllers.

Note the magnitude flatness of sensitivity function *S* produced by the fractional-order PID controller, which implies that it attenuates better disturbances/perturbations for a wider frequency band. On the other side, complementary sensitivity function *T* successfully attenuates high-frequency noise. Thus, a better disturbance/perturbation and noise rejection characteristic is obtained with the fractional-order PID controller approximation and therefore, the closed-loop system will exhibit a robust performance.

In the following section, a generation of the fractional-order PID approximation to facilitate controller implementation is derived. Experimental results are also provided and described in that section.

### 3.2. Generalization of Controller for the Implementation

Since the converter of Figure 1 operates in buck and boost conversion modes depending on the value of duty cycle *D*, a general structure for the controller must be determined. The objective is to investigate and determine if the proposed controller appropriately regulates voltage in either conversion mode.

Electrical implementation of the controller (16) requires a simpler mathematical representation. By using the partial fraction expansion of (16), one obtains mathematical expressions whose electrical equivalence is standard and well known. Before synthesizing the electrical arrangement that describes the controller (16), it is necessary to determine the type of roots that will be obtained in a general way. Therefore, by considering the effect of $T_{i}$ previously described in Figure 4, the controller will be given by (16), $G_{c}\left(s\right)=k_{c}N\left(s\right)/D\left(s\right)$ ($T_{i}\to \infty$) or $G_{c}\left(s\right)=k_{c}D\left(s\right)/N\left(s\right)$ ($T_{i}\to 0$).

**Figure 8:**
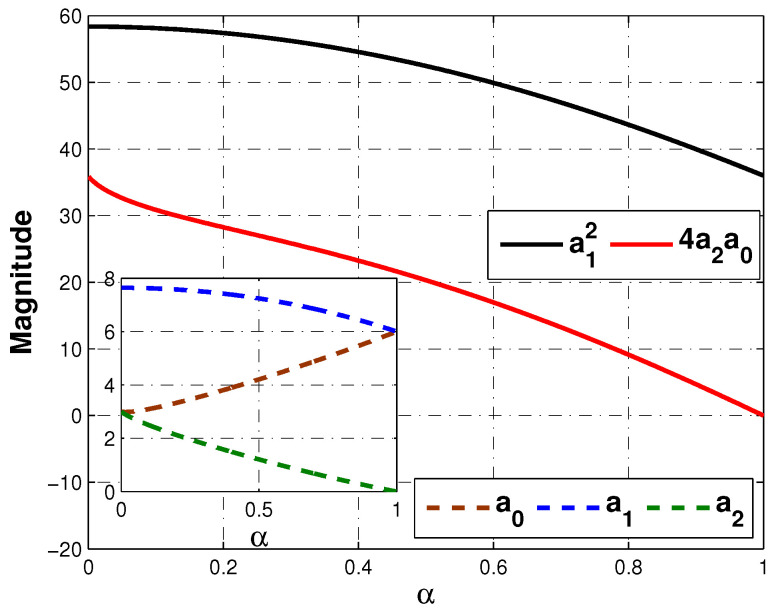
Approximation parameters $a_{0}\left(\alpha \right)$, $a_{1}\left(\alpha \right)$, $a_{2}\left(\alpha \right)$ and values $a_{1}^{2}$, $4a_{2}a_{0}$ that ensure $a_{1}^{2}>4a_{2}a_{0}$.

Please note that in all cases, the controller $G_{c}\left(s\right)$ depends on N(s) and D(s), which are numerator and denominator of approximation $s^{\alpha }$. Since both N(s) and D(s) of approximation (9) are quadratic polynomials, as long as $a_{1}^{2}>4a_{2}a_{0}$, the roots of controller $G_{c}\left(s\right)$ will be real. Knowing that $a_{0}$, $a_{1}$ and $a_{2}$ depend on 0<α<1, Figure 8 proofs that condition $a_{1}^{2}>4a_{2}a_{0}$ holds for every value of α, therefore, the partial fraction expansion of $G_{c}\left(s\right)$ will be given in terms of real poles only as follows,

(18) $$G_{c}\left(s\right)=\left(\frac{A_{1}}{\gamma_{1}s+1}\right)+\left(\frac{A_{2}}{\gamma_{2}s+1}\right)+\left(\frac{A_{3}}{\gamma_{3}s+1}\right)+\left(\frac{A_{4}}{\gamma_{4}s+1}\right)+A_{5}.$$

**Figure 9:**
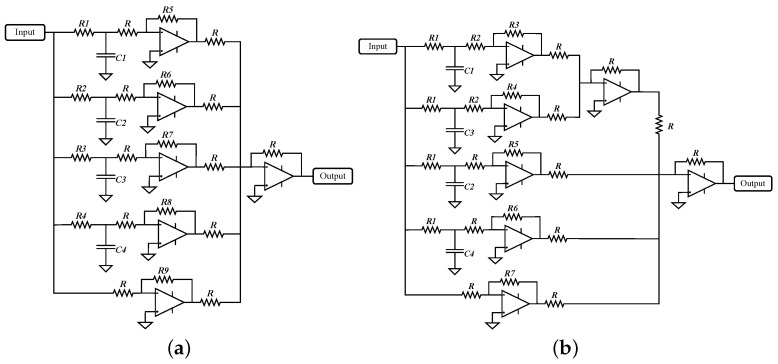
(**a**) Electrical representation of partial fraction expansion of controller (18). (**b**) Electrical representation of controller (18) for parameter values of Table 5.

Since the first four terms resemble an RC circuit transfer function, the partial fraction expansion of the controller (18) can be directly generated through RC circuits and OPAMPs as inverting amplifiers and in adder configuration. In Figure 9a, the electrical arrangement to implement the controller (18) is shown. Gamma coefficients are the equivalence of multiplying $R_{1}C_{1}$ to $R_{4}C_{4}$, and constants *A*’s are the gains of inverting amplifiers obtained by dividing $R_{5}$ to $R_{9}$ over *R*.

Constant values to represent fractional-order PID controller approximation (17), whose coefficients are given in Table 3 for buck and boost conversion modes, in its partial fraction expansion (18) are shown in Table 5 columns 1 to 3. Due to resulting gain values for the controller (18), the electrical circuit of Figure 9a is rearranged to consider the sign of $A_{1}$ and $A_{3}$, thus resulting in the electrical circuit of Figure 9b, whose parameter values are provided in Table 5 columns 4 to 6.

Note from Table 5 that the value of constants $A_{1}$ and $A_{3}$ for both conversion modes are very small and can be neglected. For this reason, the top part of electrical circuit for the controller in Figure 9b can be also omitted in the implementation with no effect in the final result, since its contribution is in the range of ηV.

**Figure 10:**
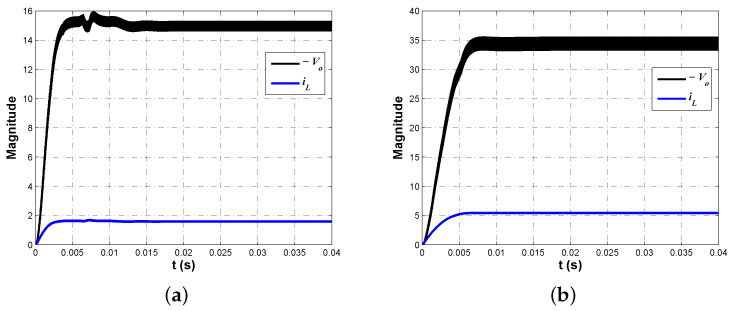
Regulated output voltage $V_{o}$ and inductor current $i_{L}$ of converter in Figure 1 for (**a**) buck and (**b**) boost conversion modes.

Using PSIM 9.0 (Powersim Inc., 2001-2010), the proposed arrangement was tested through the electrical simulation of the complete system. In Figure 10, the output voltage $V_{o}$ and inductor current $i_{L}$ for converter in Figure 1 are shown. Synthesized controllers effectively regulated output voltage in both buck $V_{o}=15$ V (Figure 10a) and boost $V_{o}=35$ V (Figure 10b) conversion modes, while operating the converter in continuous conduction mode, as can be corroborated through inductor current.

On the other hand, implementation results confirmed the viability and effectiveness of the proposed approach. The components for the experiment are all commercial and were obtained from Mouser Electronics, Mexico. The experiment technical characteristics are the following: a very high current capacity inductor 1140-103K-RC of 10 mH with ±20%, a DC resistance (DCR)of 2.76 Ω and 10 A. A polypropylene metalized film capacitor of 30 μF with max${}_{\mathrm{DC}}$ voltage of 500 V, tolerance of 5% and equivalent series resistance (ESR)of 3.5 mΩ. A high current capability power MOSFET NTP5864NG with maximum drain-to-source voltage of 60 V, continuous drain current of 63 A and $R_{\mathrm{ON}}=12.4$ mΩ. Lastly, a diode SR504 R0 with forward voltage of 0.55 V. The controller was implemented with the high speed, 4 MHz wide bandwidth quad junction field effect transistor (JFET) inputs operational amplifier LF347N and the pulse width modulation (PWM) signal was created with the traditional TL494. Capacitors and resistances of described values with tolerances of ±5% and ±1%, respectively.

**Figure 11:**
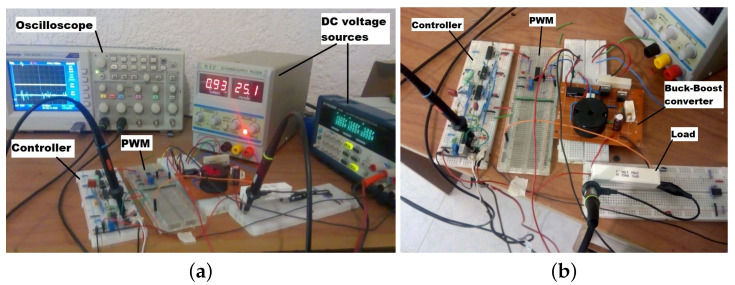
(**a**) Experiment table with oscilloscope, DC voltage sources and the electrical system. (**b**) Electrical system composed of fractional-order controller approximation, pulse width modulation (PWM) generator and buck–boost converter with the corresponding load.

In Figure 11 evidence of the experiment table is shown. From left to right are the oscilloscope, the fractional-order controller approximation, the PWM generator, DC voltage sources and the buck–boost converter with the corresponding load.

**Figure 12:**
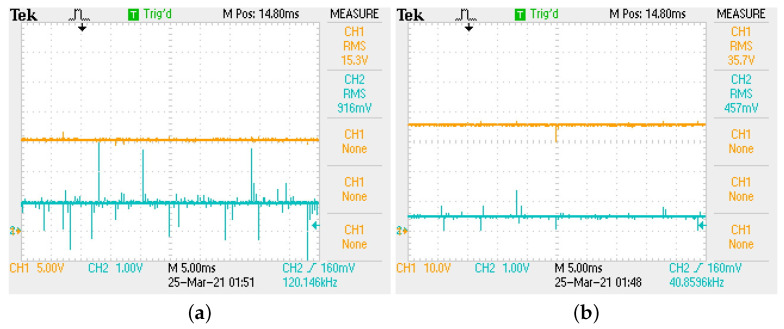
(**a**) Regulation to $V_{o}=15$ V in buck mode. (**b**) Regulation to $V_{o}=35$ V in boost mode.

In Figure 12 the voltage regulation of buck–boost converter can be corroborated in both conversion modes. In Figure 12a,b the output voltage (top signal) corroborates buck mode ($V_{o}=15$ V) and boost mode ($V_{o}=35$ V), respectively.

**Figure 13:**
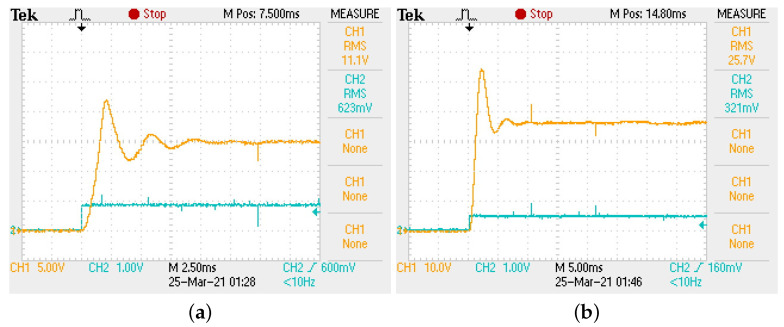
Tracking characteristic of buck–boost converter. (**a**) Buck mode. (**b**) Boost mode.

In Figure 13 the tracking characteristic of buck–boost converter is shown. As can be seen, the controller successfully regulates output voltage with a fast and stable tracking characteristic. It is important to mention the similarity of implementation results with those predicted through Figure 5 and Table 4, column 2, since boost mode regulation exhibits a faster response compared to the one produced in the buck mode.

## 4. Discussion

In this paper, the effectiveness and viability of a fractional-order PID controller approximation to regulate voltage in a buck–boost converter were addressed. The importance of the converter rests in its variety of applications from which biomedicine ones take on special relevance, since the need for a stable and fast-response source of power is one of the main drawbacks of emerging cardiac technologies, which as in high-power systems, require an appropriate energy management/harvesting/storage strategy.

From the proposed approach, the controller design method considered both performance of closed-loop response and robustness. A biquadratic module that exhibits flat phase response was used to generate the controller structure. Fractional calculus is integrated due to its proven ability to describe systems with higher accuracy and robustness against parameter variations/uncertainties.

The proposed approach viability is investigated as an alternative for highly efficient converters such as Silicon-Carbide ones. Experimental results confirmed effectiveness of the controller to regulate output voltage in a buck–boost converter using a single control loop. These results open the possibility of applying this approach to a current control mode to determine if regulation velocity can be enhanced even more.

Although it could represent a disadvantage the required extra hardware for the implementation of the proposed controller, this could be dismissed considering the promising results of the proposed approach as well as the availability in commercial values of the extra components.

## 5. Conclusions

In this paper, a fractional-order PID controller approximation was suggested to regulate voltage in a buck–boost converted. The model of the system was linearized through the small-signal technique around the equilibrium point. The resulting linearized plant was divided into minimum and non-minimum phase parts, which simplified the controller design, allowing us to focus it on the minimum phase part only. The controller design considered both performance and robustness, while a simple tuning method allowed us to determine directly appropriate gains to achieve stability margin requirements. Synthesized controllers effectively regulated voltage in both conversion modes with a stable, fast, and good tracking characteristic. Superior disturbance/perturbation and noise rejection characteristic was also determined.

By comparing performance parameters of typical PID controller with those obtained by the fractional-order PID approximation allowed us to determine quantitatively the proposed approach superiority in time and frequency domains. To corroborate the experimental viability, a generalization for the controller structure was derived. The resulting electrical arrangement is implementable easily through RC circuits and OPAMPs. Experimental results confirmed the proposed controller effectiveness and its viability, since the parameter values are all commercial.

Lastly, it is worth mentioning that numerical simulations not only predicted effectiveness of the controller but its actual effect, since the obtained experimental results confirmed data of Figure 5 and Table 4, column 2 for both conversion modes.

## Figures and Tables

**Table 1:** Parameter values of buck–boost converter in Figure 1.

Element	Notation	Value
DC voltage source	$V_{g}$	25 V
Capacitor	*C*	30 μF
Inductor	*L*	10 mH
Resistance	*R*	10 Ω
Switching frequency	$f_{s}$	20 kHz

**Table 2:** Values of $\phi_{m}$, $\phi_{pm}$ and α for buck/boost conversion modes.

Parameter	Notation	Buck	Boost
Phase margin	$\phi_{m}$	90.7${}^{\circ }$	90.5${}^{\circ }$
Uncontrolled plant phase	$\phi_{pm}$	$-89.3^{\circ }$	$-89.5^{\circ }$
Fractional-order	α	0.6745	0.6727

**Table 3:** Coefficients for approximation of the fractional-order PID controller (17).

Coefficient	Buck	Boost
$\rho_{1}$/$\psi_{1}$	$9.866\times 10^{5}$/$5.729\times 10^{5}$	$3.434\times 10^{6}$/$1.996\times 10^{6}$
$\rho_{2}$/$\psi_{2}$	$2.798\times 10^{11}$/$5.694\times 10^{10}$	$3.391\times 10^{12}$/$6.941\times 10^{11}$
$\rho_{3}$/$\psi_{3}$	$1.798\times 10^{16}$/$1.629\times 10^{15}$	$7.607\times 10^{17}$/$6.954\times 10^{16}$
$\rho_{4}$/$\psi_{4}$	$3.321\times 10^{20}$/$8.092\times 10^{18}$	$4.907\times 10^{22}$/$1.214\times 10^{21}$
*k*	0.4714	0.4749

**Table 4:** Closed-loop response performance parameters for buck/boost conversion modes, where $e_{ss}$, τ, $t_{r}$, $t_{p}$, $t_{s}$ and %*M* stand for steady-state error, time constant, rising time, peak time, settling time and overshoot, respectively.

Notation	FOPID	PID 1st Option	PID 2nd Option
$e_{ss}$	0	0	0
τ	10.6/3.03 μs	99.8/84.2 μs	98.4/8.27 μs
$t_{r}$	11.6/3.31 μs	113/93.8 μs	99.5/8.29 μs
$t_{p}$	30/8.55 μs	299/245 μs	286/21.9 μs
$t_{s}$	134/38.3 μs	1.71/1.36 ms	2.94/0.32 ms
%*M*	47%	52/50%	69/74%
$\phi_{dm}$	60${}^{\circ }$	60${}^{\circ }$	30${}^{\circ }$

**Table 5:** Constants *A*’s and γ’s of controller (18) and parameter values for electrical arrangement of Figure 9b for buck and boost conversion modes.

Constant	Buck	Boost	Element	Buck	Boost
$A_{1}$	$-1.7675\times 10^{-5}$	$-1.7523\times 10^{-5}$	$R_{1}$	100 Ω	10 Ω
$A_{2}$	1.1981	1.2018	$R_{2}$	100 kΩ	100 kΩ
$A_{3}$	$-1.1981\times 10^{-6}$	$1.2018\times 10^{-6}$	$R_{3}$	1.76 Ω	1.75 Ω
$A_{4}$	17.6752	17.5233	$R_{4}$	0.12 Ω	0.12 Ω
$A_{5}$	0.4714	0.4749	$R_{5}$	1.2 kΩ	1.2 kΩ
$\gamma_{1}$	$2.19\times 10^{-6}$	$0.631\times 10^{-6}$	$R_{6}$	17.7 kΩ	17.5 Ω
$\gamma_{2}$	$14.14\times 10^{-6}$	$4.04\times 10^{-6}$	$R_{7}$	471 Ω	475 Ω
$\gamma_{3}$	$24.86\times 10^{-6}$	$7.11\times 10^{-6}$	*R*	1 kΩ	1 kΩ
$\gamma_{4}$	$160.22\times 10^{-6}$	$45.51\times 10^{-6}$	$C_{1}$	0.022 μF	0.063 μF
			$C_{2}$	0.142 μF	0.404 μF
			$C_{3}$	0.25 μF	0.712 μF
			$C_{4}$	1.602 μF	4.55 μF
